# Assessment of the Effect of Wearing a Surgical Face Mask on Tear Film in Normal Eye Subjects

**DOI:** 10.1155/2022/2484997

**Published:** 2022-08-16

**Authors:** Mana A. Alanazi, Gamal A. El-Hiti, Rashid Al-Tamimi, Abdullah M. Bawazir, Essam S. Almutleb, Raied Fagehi, Saud A. Alanazi, Ali M. Masmali

**Affiliations:** Department of Optometry, College of Applied Medical Sciences, King Saud University, Riyadh 11433, Saudi Arabia

## Abstract

**Purpose:**

To assess the effect of wearing a face mask for a short time on the tear film parameters in normal eye subjects.

**Methods:**

Fifty-four normal eye subjects (14 female and 40 male) aged 18–40 years (23.8 ± 4.4 years) were recruited. A standardized patient evaluation of eye dryness was completed first, followed by noninvasive tear break-up time, phenol red thread, and tear ferning tests. A 5-minute gap was allowed between the tests. The subjects were asked to wear a surgical mask for one hour. The measurements were taken both before wearing a face mask and immediately after its removal.

**Results:**

Significant (Wilcoxon test) differences were found between the standardized patient evaluation of eye dryness (*p*=0.002) and the noninvasive tear break-up time scores (*p* < 0.001) before and after wearing face masks. No significant differences (Wilcoxon test, *p* > 0.05) were found between the phenol red thread scores and tear ferning grades before and after wearing face masks. Strong correlations (Spearman's rank correlation coefficient, *r*) were found among the standardized patient evaluation of eye dryness score (*r* = 0.590; *p* < 0.001), noninvasive tear break-up time measurements (*r* = 0.631; *p* < 0.001), and the tear ferning grades (*r* = 0.517; *p* < 0.001) before and after wearing the mask. A medium correlation (*r* = 0.377; *p*=0.005) was found between the noninvasive tear break-up time scores and tear ferning grades before wearing the mask.

**Conclusions:**

Wearing a surgical face mask for a short duration of one hour has an effect on ocular tear film in normal eye subjects. Dry eye symptoms and tear break-up increased after wearing a face mask compared with those experienced before wearing one.

## 1. Introduction

Tear film stability is vital for vision and a healthy ocular surface. Changes in tear film thickness cause aberrations in the optical system [1]. Tear film can be assessed using a variety of tools, including noninvasive tear break-up time (NITBUT) [2], phenol red thread (PRT) [3], Schirmer [3], osmolarity [4], tear meniscus height (TMH) [5], tear ferning (TF) [6], tear evaporation rate (TER) [7], and others along with dry eye questionnaires [8]. These tools are designed for either research purposes or clinical examination use [9]. These tests can assess the conditions that might lead to symptoms of dry eye, serious damage to the ocular surface, and vision deterioration [9].

Dry eye is a complex disorder that affects the ocular surface and originates due to several factors [10–12]. Dry eye is associated with a loss of tear film stability and homeostasis, ocular symptoms, and hyperosmolarity [13]. The tear film structure is complex, and its main components are water, lipids, and mucins [14]. The aqueous content is produced by lacrimal glands, and the lipids are produced by meibomian glands. Mucins are produced by the lacrimal glands and conjunctival goblet cells and regulate the surface tension and facilitate the spread of tear film over the cornea [14]. The risk factors for dry eye may vary but are mainly due to topical and systemic medications, skin diseases, ophthalmic surgery, ocular allergies, chemicals, computer and screen use, vitamin deficiencies, contact lenses, environmental factors, and aging [12]. Environmental and mechanical factors can lead to surface alterations leading to blepharitis [15]. The causative factors for blepharitis could also include chronic parasitic or bacterial ocular surface infections and skin inflammation. In fact, the exact pathophysiological mechanism of blepharitis is not well-known and is multifactorial. Blepharitis leads to inflammation and discomfort of the ocular surface and progresses to dry eyes [16, 17]. The prevalence of dry eye ranges from 5 to 50% of the world population, in which women are more likely to experience dry eye than men [18]. The two most common types of dry eye are evaporative and aqueous deficiency, due to the dysfunction of meibomian and lacrimal glands, respectively [10]. Dry eye symptoms include burning, grittiness, tearing, pain, redness, blurry vision, dryness, and tired eyes. Therefore, a regular evaluation of the ocular surface is important to avoid problems associated with dry eye.

As COVID-19 has become a pandemic, the most effective way to reduce its spread continues to be wearing a face mask. Therefore, wearing a face mask is now ubiquitous, particularly in crowded places. As part of the impact of wearing a face mask on our daily lives, it is necessary to investigate its effect on the tear film. A recent study conducted among individuals wearing a face mask for a long duration in a medical practice suggested a relationship between wearing a mask and ocular tear film instability [19]. The ocular surface disease index (OSDI) was used to assess symptoms associated with dry eyes. Symptoms of irritation and inflammation on the ocular surface have been experienced by face mask wearers [19].

The present study assesses the impact of wearing a face mask for a short duration on the quality and quantity of tears in normal eye subjects.

## 2. Subjects and Methods

Fifty-four healthy participants free of ocular surface disease (14 females and 40 males) aged 18–40 years (23.8 ± 4.4 years) were recruited to test the effect of surgical masks on the tear film. In addition, 50 normal-eye subjects (15 females, 35 males; 22.9 ± 4.1 years) participated in the study as a control group in which no mask was worn. A slit lamp was used to check for abnormalities within the eyelids, lashes, and meibomian glands. The exclusion criteria included subjects with thyroid disorders, a high body mass index (>25 kg/m^2^), a high cholesterol level (>4 mmol/L), a high refractive error, vitamin A and D deficiencies, hypertension, anemia, diabetes, and smokers. In addition, subjects with a history of ocular surgery, contact lens wearers, and pregnant or breastfeeding women were excluded from the study. Written informed consent was obtained from each subject before conducting the research. The subjects were treated according to the Helsinki declaration. A SPEED questionnaire was completed first, followed by the NITBUT, PRT, and TF tests. A 5-minute gap was allowed between the tests.

A three-layer surgical face mask (The Band Med, China) was used in the present study. The mask contains an invisible metal strip to fit snugly around the nose. The subjects were asked to wear the mask for one hour. Measurements were taken both before wearing the face mask and immediately after its removal. For comparison, the measurements were performed twice with a one-hour gap in the right eye of the subjects in the control group.

The SPEED questionnaire was developed to assess the severity of dry eye symptoms over time and has good validity and consistency [20–22]. The questionnaire contains three sections that are associated with the presence or absence, frequency, and severity of dry eye symptoms. It also has three different timeframes (now, the last 72 hours, and the last 3 months). Questions on dry eye symptoms, frequency, and severity are answered on a scale from 0 to 4. The final score ranges from 0 to 28. A score from 0 to 4, 5,8, and more than 8 indicates mild, moderate, and severe dry eye symptoms, respectively [23].

The NITBUT test was performed using EASYTEAR®view+ (EASYTEAR S.R.L., Via Maioliche, Trento, Italy). The NITBUT was recorded as the number of seconds that elapsed between the last blink and the appearance of the first dry spot in the tear film [24].

The PRT test was performed using a cotton thread (Zone-Quick, Showa Yakuhin Kako Co., Ltd., Tokyo, Japan) containing a pH indicator. The PRT thread with a 3 mm bent was inserted in the lower fornix, then the subject closed the eye. The thread was removed after 15 seconds and the length of the colored portion was measured in mm [3].

A small tear sample (1 *μ*L) was collected from the lower meniscus of the right eye using a glass capillary tube (10 *μ*L) purchased from Merck (Schnelldorf, Germany). The tear sample was dried for 10 min at 23°C with a humidity of 15%. An Olympus DP72 digital microscope (Tokyo, Japan) was used to observe and capture the TF images (magnification power of 10x). The TF patterns were graded based on the five-point TF grading scale with 0.1 increments [25].

## 3. Statistical Analysis

Microsoft Excel 2010 (Microsoft Office, Microsoft Corp., Redmond, WA, USA) was used to collect the data. The Statistical Package for the Social Sciences software (SPSS; IBM Software, version 23, Armonk, NY, USA) was used to analyze the data. A correlation coefficient was used to test the association between different parameters [26]. The data collected from 54 normal eye subjects (23.8 ± 4.4 years) were not normally distributed (Kolmogorov–Smirnov, *p* < 0.05). Therefore, the median scores of the SPEED questionnaire and the NITBUT, PRT, and TF tests were represented by their median (interquartile range; IQR).

## 4. Results

The median (IQR) for the SPEED, NITBUT, PRT, and TF scores recorded in the study group are summarized in Table 1. For the control group, there were no significant differences between the two scores obtained from the SPEED questionnaire and the NITBUT, PRT, and TF tests that were recorded with one-hour gaps. The data collected before and after wearing a face mask were different. Significant (Wilcoxon test) differences were found between the SPEED scores (*p*=0.002) and the NITBUT measurements (*p* < 0.001), before and after wearing a face mask. No significant (Wilcoxon test, *p* > 0.05) differences were found between the scores obtained from the PRT and TF tests before and after wearing a face mask.

The SPEED score decreased after wearing a face mask in 18 subjects (33.3%), increased in five (9.3%), and remained unchanged in the majority (*n* = 31; 57.4%). For the NITBUT test, the score decreased in the majority of subjects (*n* = 38; 70.4%), increased in 13 (24.1%), and remained unchanged in three individuals. The PRT score decreased in 23 (42.6%), increased in 24 (44.4%), and remained unchanged in 7 (13%) subjects. Based on the TF test, the quality of tears decreased in the majority of subjects (*n* = 30, 55.6%) and improved in the remaining individuals (*n* = 24; 44.4%).

**Figure 1:**
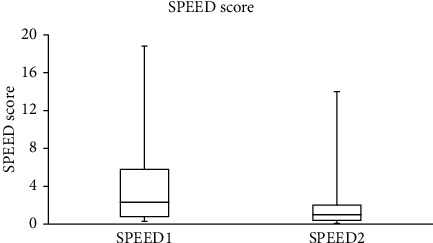
Side-by-side boxplots for the SPEED scores for the study group before (SPEED1) and after wearing (SPEED2) a face mask.

**Figure 2:**
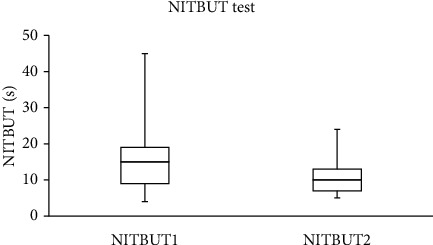
Side-by-side boxplots for the NITBUT scores for the study group before (NITBUT1) and after wearing (NIBUT2) a face mask.

**Figure 3:**
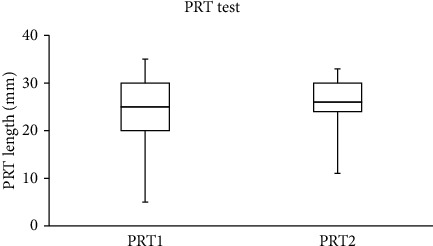
Side-by-side boxplots for the PRT scores for the study group before (PRT1) and after wearing (PRT2) a face mask.

**Figure 4:**
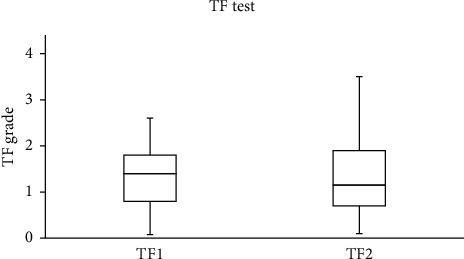
Side-by-side boxplots for the TF grades for the study group before (TF1) and after wearing (TF2) a face mask.

**Figure 5:**
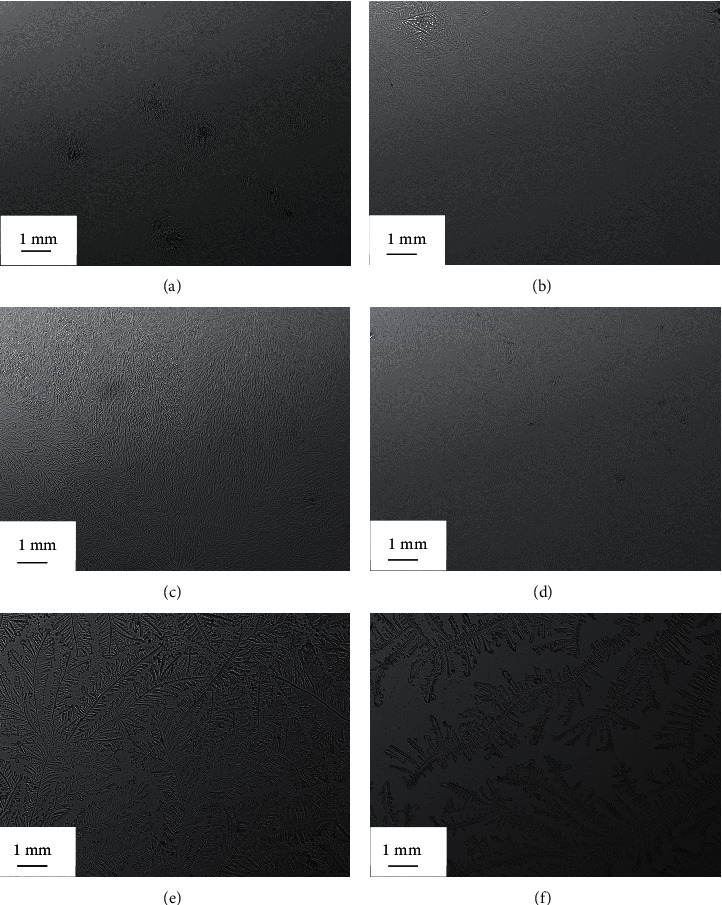
Representative TF images for the tears of three subjects before (a, c, e)and after (b, d, and f) wearing a face mask.

The side-by-side boxplots for the SPEED questionnaire and the NITBUT, PRT, and TF scores for the study group, before and after wearing a face mask, are represented in Figures 1–4. The TF images of the dried tears collected from three subjects before (*a*, *c*, and *e*, respectively) and after wearing (*b*, *d*, and *f*, respectively) a face mask are shown in Figure 5.

Strong correlations (Spearman's rank correlation coefficient, *r*) were found between the SPEED score (*r* = 0.590; *p* < 0.001), the NITBUT measurements (*r* = 0.631; *p* < 0.001), and the TF grades (*r* = 0.517; *p* < 0.001) before and after wearing a face mask. A medium correlation (*r* = 0.377; *p*=0.005) was found between the NITBUT and TF scores before wearing the mask.

## 5. Discussion

The present study suggests that wearing a surgical face mask for a short time of one hour has an effect on tear film stability based on the scores recorded by the SPEED questionnaire and the NITBUT test. However, no significant effect was found on tear volume measured by the PRT test and the quality of tears determined by the TF test.

A study conducted between January 2020 and August 2020 in the US showed that chalazion incidence increased significantly as a result of wearing a face mask compared with the data recorded between 2016 and 2020 [27]. Wearing a mask induces increased TER and dry eye symptoms [19, 28]. A survey was conducted among 107 medical students in Italy (average age = 28.5 years; 69 females and 36 males) to assess the effect of COVID-19 on dry eye symptoms. The average OSDI score was 21, and 57% of the subjects scored 15 or more [19]. Ocular surface discomfort was experienced by 10.3% of the subjects (*n* = 11). In addition, 19.6% of the subjects (*n* = 21) used artificial tears daily [16]. Dry eye symptoms are relatively common in patients with COVID-19, which therefore may affect the ocular tear film [28, 29].

The use of OSDI among subjects with a history of dry eyes (*n* = 203) showed an association between dry eye symptoms and wearing a surgical face mask for a minimum of three hours [30]. Males were found to have significantly (Mann–Whitney *P*=0.004) lower OSDI scores compared with females [30]. However, another online survey (*n* = 1219) using the OSDI claimed no significant association between dry eye symptoms and wearing a face mask [31].

Ocular surface irritation is associated with wearing of face mask among normal individuals [32]. Wearing a face mask likely causes air to blow around the eyes, leading to discomfort and irritation. The air blowing tends to increase TER and therefore leads to the inflammation and irritation of the tear film [19, 33, 34]. Air convection also contributes to dry environments. Indeed, dry eye incidents are increasing with the use of some powered respirators with air-purification systems [35]. In addition, the use of chemical protecting hoods leads to dryness with contact lenses and irritation due to air blowing inside the mask [36]. The use of a continuous positive airway pressure mask leads to eye irritation as a result of air leakage [37–39]. It is believed that wearing a face mask leads to frequent touching of the eye and rubbing as a result of discomfort due to air blowing into the eye.

Long-term wearing of a face mask, which is more likely during the present COVID-19 pandemic, could cause ocular surface discomfort [40, 41]. COVID-19 has an impact on our daily lives and the tear film. For example, the number of eye surgeries dropped in Italy during the COVID-19 lockdown [42]. Patients with coronavirus 2 showed eye burning, tearing, foreign body sensation, conjunctival hyperemia, and signs of blepharitis [43]. In addition, COVID-19 infection could lead to dry eyes and the loss of smell and taste [44]. Dry eye is associated with blepharitis, in which *Staphylococcus aureus* has been isolated [45]. Eye infection from a pathogen can be caused by droplets from coughing, sneezing, and talking [46]. Therefore, eyeglasses or goggles provide a barrier against microorganisms but lead to inflammation. Although wearing a face mask has an effect on the ocular tear film, it remains an effective way to reduce the spread of COVID-19. Practitioners can provide help and advice to reduce or treat symptoms associated with dry eye [47].

## 6. Conclusions

Wearing a surgical face mask for a short duration of one hour has an effect on the ocular tear film in normal eye subjects. Dry eye symptoms and tear break-up increased after wearing a face mask compared with those experienced before wearing one. However, the difference between the scores collected from the phenol red thread and tear ferning tests, before and after wearing a face mask, was not significant.

## Figures and Tables

**Table 1:** The median (IQR) for the SPEED, NITBUT, PRT, and TF scores.

Parameter	beforewearing the mask	After wearing the mask	*p* value
SPEED^*∗*^	0.5 (4.5)	0.1 (1.15)	0.002
NITBUT (s)^*∗*^	15.0 (10.0)	10.0 (16)	<0.001
PRT (mm)	25 (10)	26 (6)	0.398
TF	1.4 (1.0)	1.6 (1.2)	0.198

^*∗*^Significant difference was found before and after wearing a face mask.

## Data Availability

Data are contained within the article.
